# PD29 Protocol For Rapid Reviews And Clinical Recommendations Using Artificial Intelligence Tools And Agile Processes

**DOI:** 10.1017/S0266462325101645

**Published:** 2025-12-29

**Authors:** Leonardo Luna

## Abstract

**Introduction:**

Rapid reviews are a subset of systematic reviews developed in the past decade to maintain methodological rigor while accelerating the review process. Increasingly, artificial intelligence tools are being utilized to enhance various aspects of these reviews, ensuring greater efficiency. Typically, rapid reviews support official recommendations by medical societies or healthcare institutions.

**Methods:**

Based on the Guidelines International Network-McMaster Guideline Development Checklist and publications from prominent institutions in systematic reviews, particularly the Cochrane Collaboration, the World Health Organization, McMaster University, and the Joanna Briggs Institute, an institutional protocol for rapid reviews was developed to support the creation of institutional clinical recommendations. This protocol was subjected to internal discussions within the Health Technology Assessment Unit of the National Institute of Cardiology, external evaluation by the Guidelines Committee of the Brazilian Society of Cardiology, and was refined throughout the development of guidelines for these institutions. Agile processes and artificial intelligence tools were tested to enhance efficiency.

**Results:**

The development of one position statement, four clinical recommendations, and an institutional protocol facilitated refinement of the entire process. Weekly sprint methodology was incorporated, five artificial intelligence tools were integrated to support systematic review methods, an online storage standard was established, and a task checklist was created. These modifications enabled the completion of a rapid review in approximately three months.

**Conclusions:**

The globalization of health care and rapid system changes require medical institutions to quickly develop evidence-based recommendations. Rapid reviews accelerate access to quality knowledge. This paper highlighted tools tested and incorporated to streamline the process, ensuring efficiency while maintaining the quality and scientific rigor of the final document.

